# Conversion of a Fused or Ankylosed Hip to Total Hip Arthroplasty: Is the Direct Anterior Approach in the Lateral Decubitus Position an Ideal Solution?

**DOI:** 10.3389/fsurg.2022.819530

**Published:** 2022-02-08

**Authors:** Jiale Dong, Lingtong Kong, Siming Zhang, Xifu Shang, Jiaxing Wang, Xianzuo Zhang, Chen Zhu

**Affiliations:** ^1^Department of Orthopedics, The Affiliated Provincial Hospital of Anhui Medical University, Hefei, China; ^2^Division of Life Sciences and Medicine, Department of Orthopedics, The First Affiliated Hospital of USTC, University of Science and Technology of China, Hefei, China; ^3^Department of Orthopaedics, Shanghai Jiao Tong University Affiliated Sixth People's Hospital, Shanghai Jiao Tong University, Shanghai, China

**Keywords:** total hip arthroplasty, posterolateral approach, direct anterior approach, hip fusion, ankylosed hip

## Abstract

**Background:**

Total hip arthroplasty (THA) using the direct anterior approach (DAA) is becoming increasingly popular due to its potential benefits over the posterolateral approach (PLA). However, few studies have compared the efficacies of these two surgical approaches in hip fusion treatment. This study compared early clinical direct anterior and posterolateral THA outcomes in hip fusion treatment.

**Methods:**

Here, 127 hips (65 DAA, 62 PLA) were retrospectively evaluated. Early postoperative functional outcomes of DAA and PLA groups were assessed using Harris score and Oxford Hip Score (OHS) and standard anteroposterior hip radiographs. Surgical characteristics, perioperative results, and complications within 6 months postoperatively were recorded.

**Results:**

Though baseline values were similar, Harris and OHS scores were better in the DAA group than in the PLA group at 1 and 3 months postoperatively. The average cup anteversion angle was significantly greater in the DAA group than in the PLA group (12.7° vs. 11.1°). More hips undergoing DAA were successfully orientated in both inclination and anteversion angles (46 vs. 32). Early postoperative hip function predictors were preoperative fused hip position, surgical approach, and range of motion. DAA was associated with reduced postoperative blood loss and shorter hospital stays. Furthermore, 14 vs. 8 complications occurred in the DAA vs. PLA group. Lateral femoral cutaneous nerve injuries were observed in eight hips (12.3%) of the DAA group.

**Conclusion:**

For fused or ankylosed hips, THA using DAA in the lateral decubitus position may result in excellent prosthesis positioning and faster postoperative recovery throughout early follow-up vs. PLA.

## Introduction

A fused or ankylosed hip may be a late complication of chronic inflammatory disorders or an iatrogenic result of hip fusion (1). Hip arthrodesis is a popular salvage procedure after hip tuberculosis, septic arthritis, and severe unilateral hip trauma (2, 3). Although successful hip arthrodesis provides long-term pain relief and facilitates resumption of activities involving heavy labor, range of motion loss and gait abnormalities are noted. The contralateral hip joint, ipsilateral knee joint, and spine need to be compensated accordingly, which accelerates metamorphosis (2, 3). Therefore, total hip arthroplasty (THA) is currently considered the best option due to maintenance of hip function, sparing other joints.

Although the conversion of ankylosed or fused hips to THA has proven successful throughout long-term clinical follow-ups (1, 4), the surgical technique is challenging. Surgical exposure is difficult due to the presence of surgical scars and soft-tissue contracture. It is also difficult to obtain a clear joint space by lifting or rotating the femur. Violent operations may easily cause iatrogenic fractures due to osteoporosis-related disuse. Orienting the cup prosthesis is also technically demanding because the lumbosacral joint lacks compensation during pelvic movement. Previously, the posterolateral approach (PLA) has most often been used, since exposure is clear and extension may easily be achieved (1, 5, 6).

The direct anterior approach (DAA) in THA, first described by Judet et al. is a minimally invasive neuromuscular approach involving a minimal degree of muscle injury. It is typically performed in the supine position using a tailor-made leg-traction operation table. This approach is favored among arthroplasty surgeons due to its potential advantages over other approaches: milder pain, faster recovery, better postoperative gait, and comparatively lower dislocation rate. However, there have been concerns regarding exposure difficulties due to poor femur mobility in a fused or ankylosed hip (7) that may lead to implant mispositioning, especially in procedures performed by inexperienced surgeons. To overcome these limitations, the lateral decubitus position was attempted, and preliminary success was achieved. Studies have shown that DAA-THA in the lateral decubitus position allows for superior prosthesis placement angles and satisfactory clinical results (8, 9).

At our institute, the lateral decubitus DAA in THA has been routinely used since 2016, with encouraging intraoperative and early postsurgical outcomes. However, to date, only few studies have reported clinical outcomes of lateral decubitus DAA-THA in patients with a fused or ankylosed hip. Our hypothesis is that the DAA in the lateral position can facilitate the achievement of comparable or better clinical results than the PLA. Therefore, we compared early clinical direct anterior and posterior THA outcomes in hip fusion treatment.

## Materials and Methods

### Patient Cohort

The study was conducted in accordance with the Declaration of Helsinki, with Ethics Committee approval. We retrospectively collected electronic medical records of 115 patients who underwent THA for hip fusion between January 2013 and January 2020. The inclusion criteria were (1) unilateral or bilateral hip arthroplasty for fused hip; (2) DAA approach or posterolateral approach for THA; and (3) at least 1-year of follow-up. Exclusion criteria were (1) history of lower limb nerve injury or lower limb surgery; (2) serious underlying diseases, such as severe cardio-cerebrovascular disease, liver and kidney failure; or (3) incomplete medical records.

### Surgical Technique

The surgical technique used was retrieved from medical records. All procedures were performed by the same senior surgeon. Patients were placed in the standard lateral decubitus position on an ordinary operation table.

**Figure 1:**
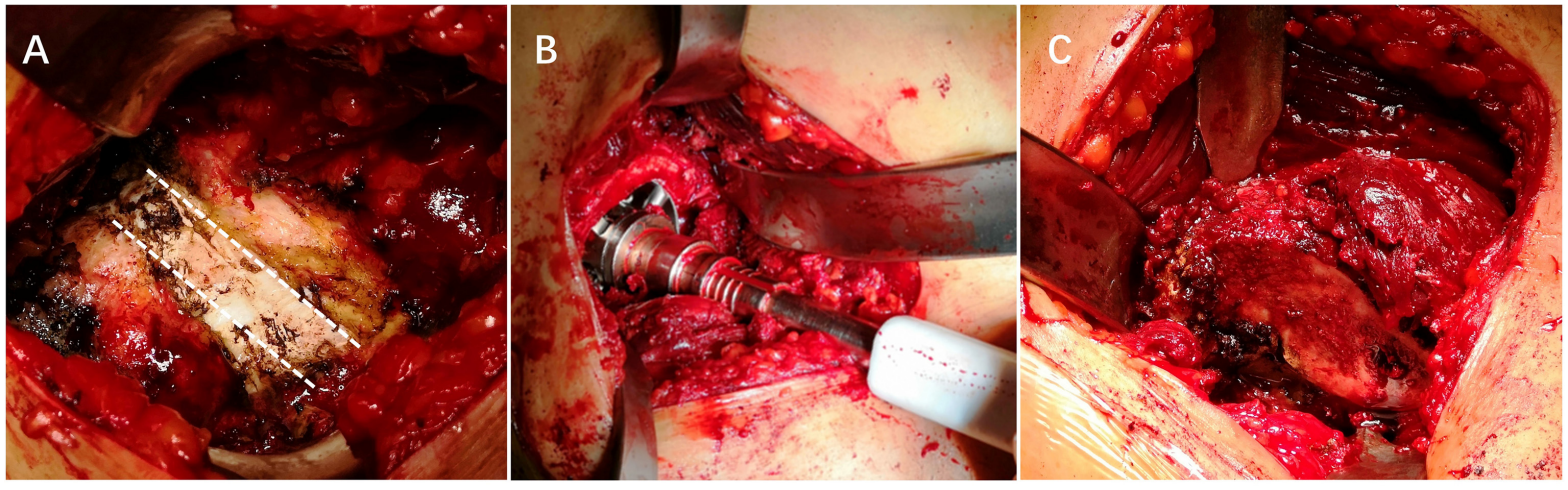
Intraoperative photographs of a fused hip. **(A)** Intraoperative photograph of the site of the fusion. **(B)** Reaming the acetabulum. **(C)** Exposing the proximal femur.

DAA-THA was conducted through Hueter's interval (Supplementary Video 4). An incision was initiated 3 cm posterolateral of the anterior superior iliac spine and extended distally 8–12 cm long toward the fibular head. Fascia was separated along the tensor fascia lata surface, and the tensor fascia lata and sartorius were passively separated inward. The ascending branches of the lateral circumflex vessels were ligated or cauterized. After releasing the rectus femoris muscle and incising the anterior joint capsule, the fused hip joint and surrounding hyperplastic osteophytes were completely visible (Figure 1). Two osteotomies of the femoral neck were performed based on preoperative templates. The femoral head, chiseled by the osteotome, was retrieved. The glenoid labrum, articular capsule, and osteophyte were removed. The acetabulum was reamed to permit the proper positioning of the cup at 35–45° inclination and 7–17° anteversion. The femur was prepared by placing the affected leg in flexion, adduction, and internal rotation (Supplementary Files Case 1). This was followed by posterolateral hip capsule excision. Sequential external circumflex muscle groups were released until the proximal femur was lifted. The femur was reamed under direct vision, and the prosthesis was implanted. Tranexamic acid (1.0 g) was applied locally, and routine closure of the incision was performed.

PLA operations were performed using the Kocher-Langenbeck approach. An arc incision was made 1 cm posterior to the greater trochanter, extending ~10 cm to the distal end. Then, the iliotibial band was split, the gluteus maximus was released, the short external circumflex muscle and joint capsule were cut, and femoral neck osteotomy was performed. Acetabular and femoral prostheses were implanted in the same manner as was described for the DAA. Similar uncemented prosthetic implants have been used in these surgeries.

### Postoperative Rehabilitation

Prophylactic antibiotics (cefuroxime) and thromboprophylaxis (low-molecular-weight heparin) were administered to all patients. Partial weight-bearing physical exercise was begun 1 day postoperatively. No hip joint movement was restricted in the DAA group, while routine hip joint precautions were indicated in the PLA group.

### Radiographic Evaluations

Standard anteroposterior hip radiographs were obtained preoperatively, immediately postoperatively, and at the last clinical visit. The inclination angle of the acetabular cup was measured using Widmer's method and the anteversion angle was determined using Lewinnek's method (10). The Danoff safety zone was used to evaluate the cups position, and a cup with 30°-50° of abduction and 5°-25° of anteversion was considered successful (11). All radiological data were evaluated by two independent radiologists.

### Functional Evaluation

Preoperative, postoperative, and follow-up statistics were collected. follow-up usually occurred at 1, 3, 6, and 12 months postoperatively and yearly thereafter. At each follow-up, joint function was assessed using Harris Hip Score (HHS) and Oxford Hip Score (OHS).

### Perioperative Characteristics

Postoperative blood loss volume was calculated based hemoglobin concentration before and after surgery via Nadler's method (12). Preoperative and postoperative leg length discrepancy were measured using full-length, lower extremity radiographs. In addition, the incidence of complications, including proximal femoral fractures, superficial incisional complications, wound infection, nerve injury, dislocation, and deep venous thrombosis, were recorded.

### Statistical Analysis

Descriptive statistics summarized characteristics and findings. Continuous variables were expressed as mean ± standard deviation, and the Kolmogorov-Smirnov normal test was used to evaluate digital sample distributions. Between-group differences were compared using the two-tailed independent sample *t*-test or Mann-Whitney *U*-test. Categorical variables were expressed as frequency and percentage, and outcomes were compared using the Chi-square or Fisher's exact tests. Linear regression equations were used to describe acetabular cup angle changes due to surgical experience. HHS after 1 month of follow-up were divided into four grades, as follows: excellent (>80 points), good (70–79 points), fair (60–69 points), and poor (50–59 points) (Supplementary Figure 1). Ordinal regression was used to analyze independent risk factors affecting postoperative HHS. The factors considered in the model included age, sex, body mass index, course of disease, etiology, fused position, preoperative HHS, surgical approach, operative time, intraoperative blood loss, prosthesis position, complications, and joint range of motion (ROM). Mediation of ROM in the relationship between fusion type and HHS at 1 month of follow-up was tested, while controlling for confounding factors (sex, age, disease course, and surgical approach). SPSS 26 software (SPSS, Armonk, NY) was used for statistical analyses, and *p* < 0.05 was considered statistically significant.

## Results

**Figure 2:**
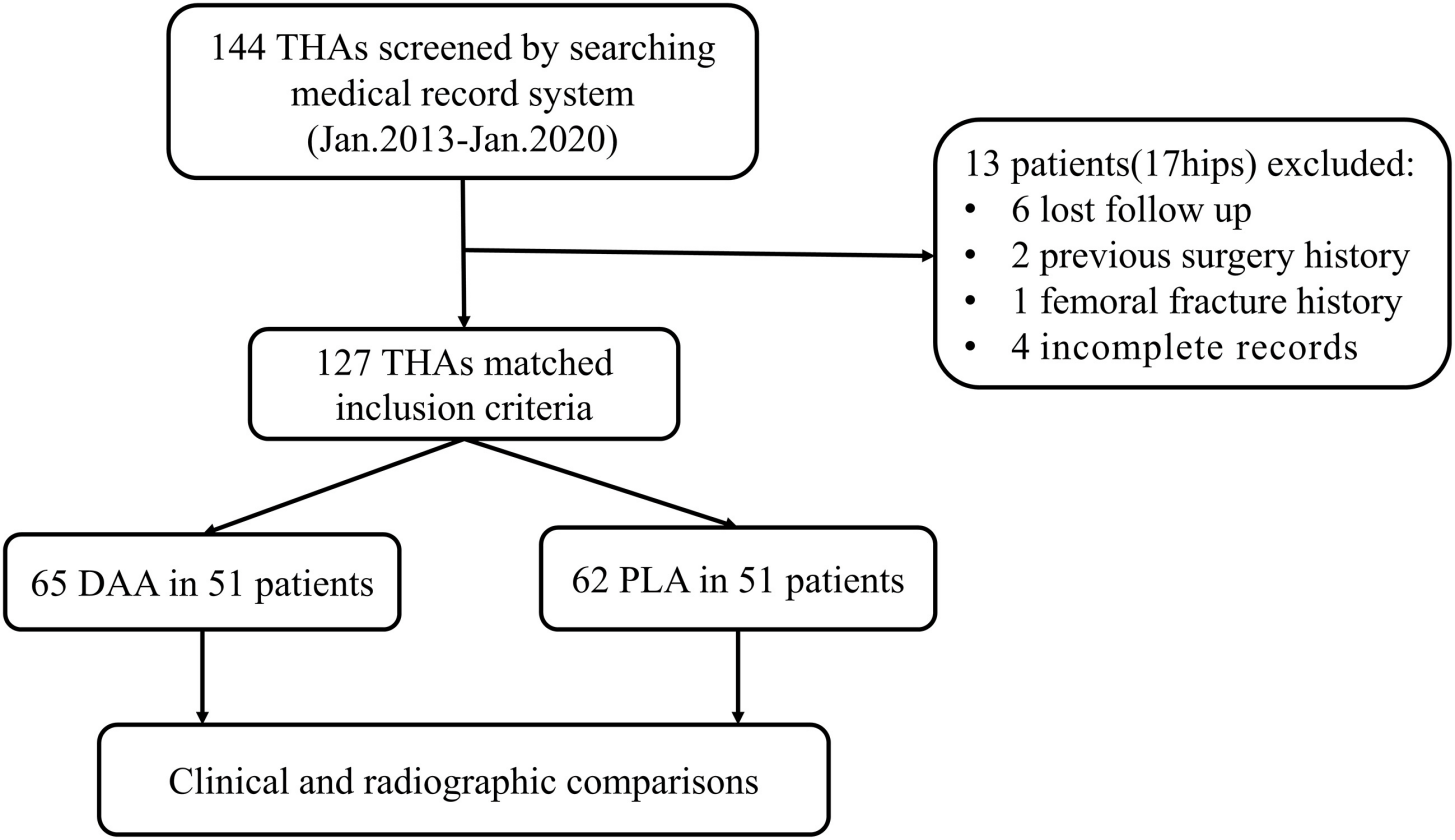
Study design and flowchart.

One hundred-two (127 hips) of 115 patients initially considered were included in the study (Figure 2). The average follow-up was 37.9 ± 18.3 months (12.0–81.2 months). Causes of hip fusion were ankylosing spondylitis and suppurative hip arthritis. Primary indications for THA were low back pain (59 cases, 46.5%), ipsilateral knee joint pain (17 cases, 13.4%), homolateral or contralateral hip pain (44 cases, 34.6%), and severe claudication (7 cases, 5.5%). Baseline characteristics of both groups were similar (Table 1).

**Table 1:** Demographics and baseline characteristics of included cases.

	**DAA**	**PLA**	***P*-Value**
	**(*n* = 65)**	**(*n* = 62)**	
Age(y)	46.0 ± 12.9	44.7 ± 11.0	0.527
Gender (Male/female)	51/14	49/13	0.937
BMI	23.5 ± 3.6	23.5 ± 4.0	0.998
Preop VAS	3.3 ± 0.6	3.2 ± 0.7	0.487
Preop HHS	44.4 ± 9.3	47.0 ± 10.2	0.141
Preop LLD	2.6 ± 1.5	2.3 ± 1.3	0.205
Causing disease	48/17	42/20	0.449
(AS/ purulent arthritis)			
Course of disease(y)			
AS	18.5 ± 9.5	15.7 ± 7.5	0.127
Purulent arthritis	39.9 ± 12.1	42.0 ± 15.2	0.646
Fuse position (Flexural/ extended)	33/32	35/27	0.521

*DAA, means direct anterior approach; PLA, posterolateral approach; BMI, body mass index; preop, preoperative; VAS, Visual Analog Scale; HHS, Harris Hip Score; LLD, Leg Length Discrepancy; preop, preoperative*.

### Functional Outcomes

**Figure 3:**
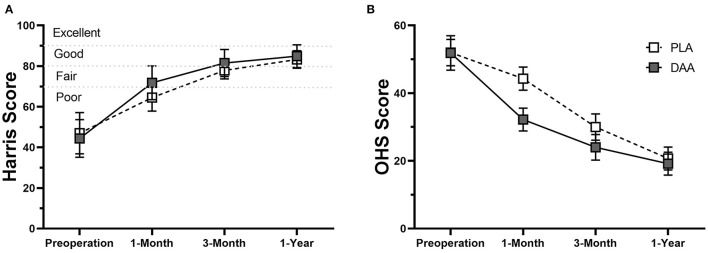
Line chart which shows the comparisons of Harris score **(A)** and OHS scores **(B)** between patients in DAA group and PLA group. The error bars indicate the standard deviation.

Objective and subjective functional status were evaluated using HHS and OHS scores. Overall, status significantly improved postoperatively (Figure 3). Mean HHS scores of the DAA group were significantly higher than those of the PLA group at 1 and 3 months postoperatively (71.8 ± 8.3 vs. 64.5 ± 6.7, *p* < 0.001; 81.6 ± 6.7 vs. 77.8 ± 4.2, *p* < 0.001, respectively). The difference was mainly attributable to feature scores. Compared to the PLA group, an increased percentage of patients in the DAA group abandoned walking aids (39 [60.0%] vs. 13 [21.0%] 1 month postoperatively, *p* < 0.001; 59 [90.8%] vs. 45 [74.2%]) at postoperative month three, *p* = 0.008) (Supplementary Table 1). One year postoperatively, no significant between-group differences were observed (85.4 ± 5.5 vs. 85.1 ± 4.3, *p* = 0.731). For 1-month to 1-year follow-up, the DAA group had better OHS scores than the PLA group (*p* < 0.05). No significant difference between-group difference in ROM was observed 1 year postoperatively (Supplementary Table 2).

### Prosthesis Information

A BetaCup (LINK, Germany) was used in 27 (21.3%) hips, a CombiCup (LINK, Germany) in 82 (64.5%), and a Trident cup (Stryker, USA) in 18 (14.2%). The bearing type included ceramic on ceramic in 106 (83.5%) hips, ceramic on polyethylene in 16 (12.6%), and metal on metal in 5 (3.9%). An LCU femoral stem (LINK) was used in 109 (85.8%) hips, whereas an Accolade stem was used 18 (14.2%).

### Radiological Outcomes

**Figure 4:**
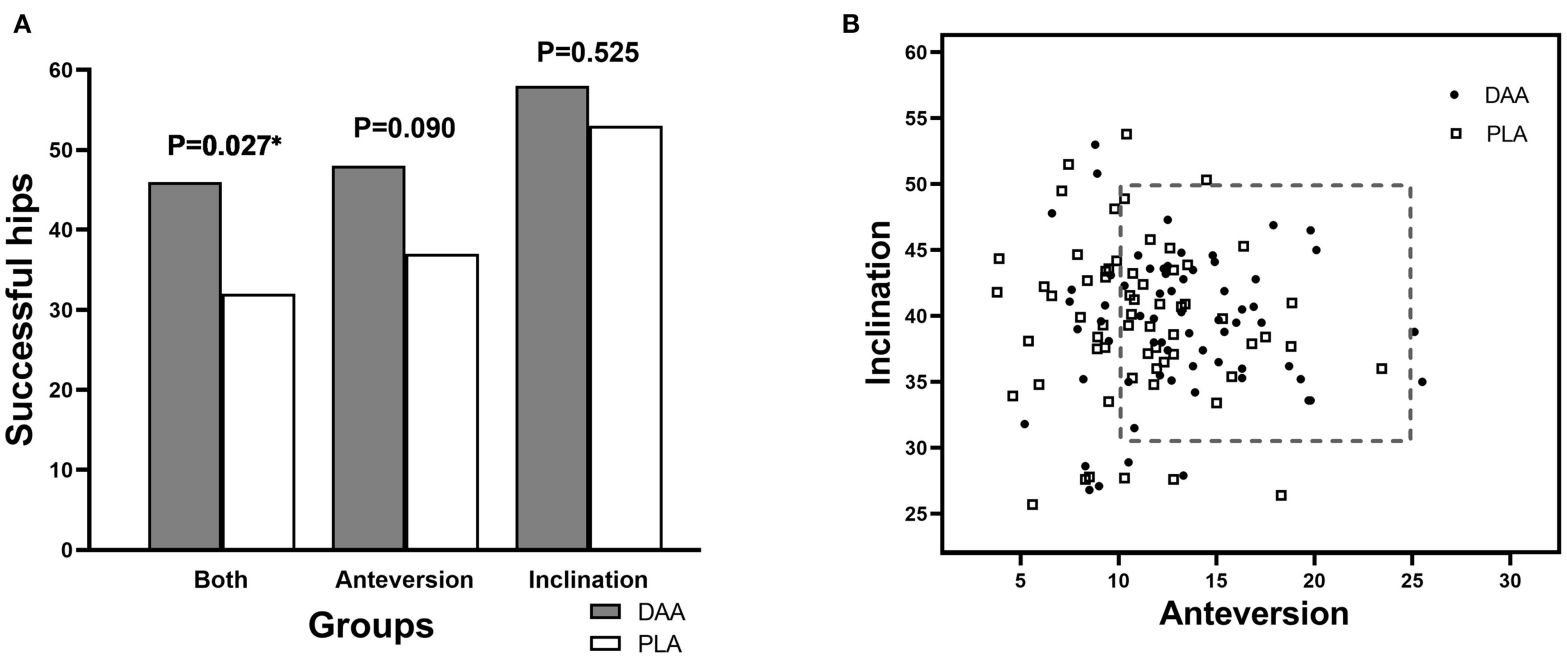
Radiological outcomes of patients in two groups. **(A)** Number of cases with successful cup orientation. **(B)** Danoff safe zone.

**Figure 5:**
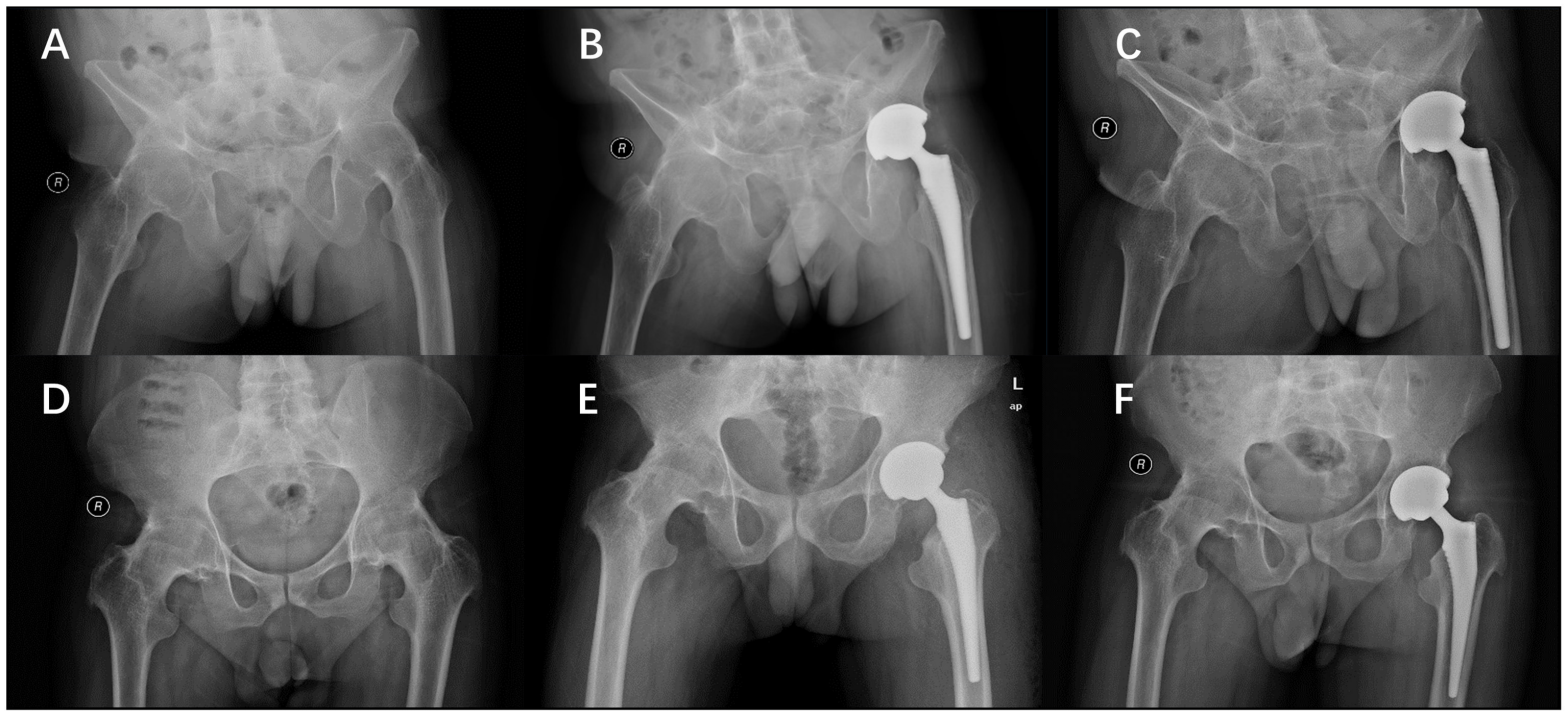
Conversion of extended hip fusion to DAA-THA in a 30-year-old male. Preoperative **(A)**, 1 month **(B)** and 1 year postoperative **(C)** radiographs. Conversion of flexural hip fusion to DAA-THA in a 33-year-old male. Preoperative **(D)**, 1 month **(E)** and 1 year postoperative **(F)** radiographs.

**Figure 6:**
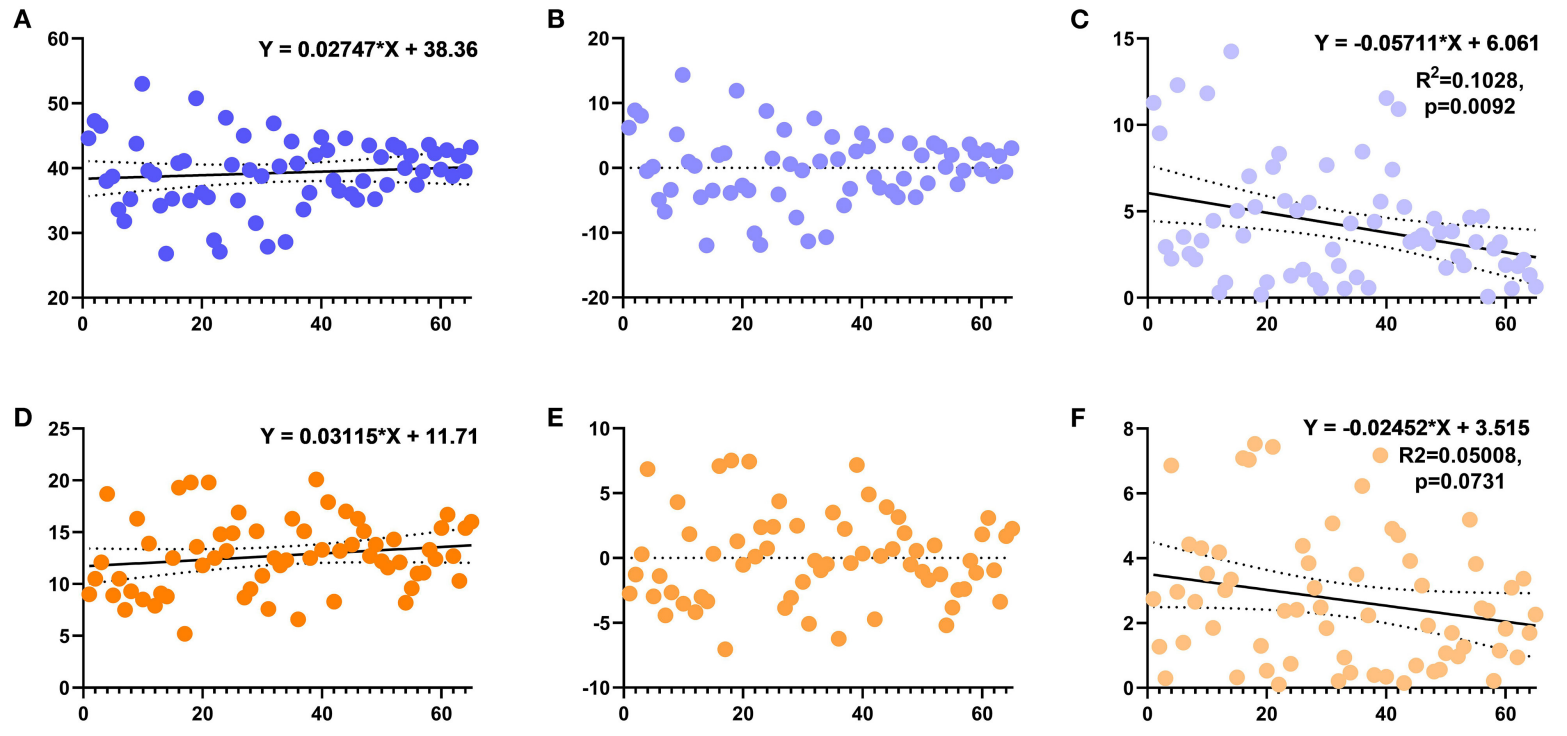
Scatter plot showing the anteversion and inclination angles of the acetabulum in the DAA group. **(A)** The inclination angle of the acetabulum changes with the learning curve and its linear regression equation (non-significant). **(B)** The residual deviation of inclination from the target. **(C)** The absolute value of the residual deviation of the acetabular inclination angle and its linear regression. **(D)** The anteversion angle of the acetabulum changes with the learning curve and its linear regression equation (non-significant). **(E)** The residual deviation of anteversion from the target. **(F)** The absolute value of the residual deviation of the acetabular anteversion and its linear regression.

The postoperative pelvic orthographic images revealed that the cup anteversion angle of the DAA group was significantly larger than that of the PLA group (12.7° vs.11.1°, *p* = 0.012). However, average cup inclination angles and femoral stem alignment did not differ (Table 2). In total, 46 hips of the DAA group (70.8%) and 32 of the PLA group (51.6%) were successfully placed within the Danoff safety zone (*p* = 0.027, Figures 4, 5). Early in the surgeon's learning curve for the DAA, inclination and anteversion acetabular cup angles periodically fluctuated around 38.36° and 11.71°, respectively, and gradually stabilized. Residual acetabular inclination angle error gradually decreased with surgical experience. Accordingly, the absolute value of residual acetabular inclination angle was linearly associated with time (β = −0.05711, R^2^ = 0.1028, *p* < 0.05, Figure 6). Acetabular anteversion deviation also seemed to decrease with as surgical experience increased. A linear regression was attempted, but results were not significant (R^2^ = 0.050, *p* = 0.073, Figure 6).

**Table 2:** Implant alignment.

	**DAA**	**PLA**	***P*-Value**
	**(*n* = 65)**	**(*n* = 62)**	
Cup alignment			
Inclination	39.3 ± 5.5	39.6 ± 6.0	0.760
Anteversion	12.7 ± 3.5	11.1 ± 3.9	0.013
Fall in the safe zone	46 (70.8%)	32 (51.6%)	0.027
Stem alignment			
Neutral	47 (72.3%)	38 (61.3%)	0.187
Varus	7	10	
Valgus	11	14	

*DAA, means direct anterior approach; PLA, posterolateral approach*.

### Postoperative Hip Joint Function

A logistic regression model identified the following risk factors for poor postoperative hip function (Table 3): preoperative fused hip position (odds ratio [OR]: 2.35, 95% confidence interval [CI]: 1.12–4.93, *p* = 0.024), surgical approach (OR: 3.11, 95% CI: 1.42–6.81, *p* < 0.01), and early postoperative ROM (OR: 1.1, 95% CI: 1.01–1.09, *p* = 0.02). It was revealed that early postoperative ROM mediated 19.8% of the total effect of fused hip position on postoperative HHS 1 month postoperatively, (Supplementary Tables 3, 4).

**Table 3:** Summary of multivariable ordinal logistic regression for 1 month follow-up.

**Factors**	***P*-value**	**Odds ratio (95%**
		**Confidence interval)**
Age(years)	0.141	1.030 (0.990, 1.072)
Gender(female)	0.158	0.469 (0.164, 1.341)
Course(years)	0.082	0.966 (0.928, 1.004)
BMI	0.594	0.975 (0.889, 1.069)
Cause (purulent arthritis)	0.838	0.872 (0.234, 3.251)
Fuse position (flexural)	0.024	2.348 (1.119, 4.926)
Preop-HHS	0.892	0.998 (0.963, 1.034)
Surgical approach (PLA)	0.005	3.110 (1.421, 6.809)
Operation time	0.320	1.005 (0.995, 1.016)
Blood Loss	0.911	1.000 (0.997, 1.002)
Cup orientation (not successful)	0.214	1.609 (0.760, 3.406)
Stem coronal alignment (not successful)	0.876	0.944 (0.456, 1.954)
Complication	0.274	1.678 (0.664, 4.237)
ROM	0.02	1.050 (1.008, 1.093)

*DAA, means direct anterior approach; PLA, posterolateral approach; BMI, body mass index; ROM, Range of Motion; HHS, Harris Hip Score; preop, preoperative*.

A comparison of early postoperative ROM and HHS was made between flexural and extended hips, which revealed that extended hips had improved ROM and HHS. Interestingly, a subgroup analysis revealed that the phenomenon was apparent only in the hips that underwent PLA surgeries (Table 4).

**Table 4:** Harris scores and ROM of patients of two groups for 1-month follow-up.

	**ROM**	***P*-Value**	**Harris**	***P*-Value**
Fused position	139.6 ± 9.7	0.003	66.3 ± 8.6	0.004
Flexural (68)				
Extended (59)	144.8 ± 9.7		70.5 ± 7.7	
(DAA)				
Flexural (33)	144.8 ± 7.4	0.558	70.2 ± 8.8	0.116
Extended (32)	146.1 ± 9.9		73.5 ± 7.6	
(PLA)				
Flexural (35)	134.6 ± 9.1	0.001	62.5 ± 6.4	0.008
Extended (27)	143.3 ± 9.3		67.0 ± 6.4	

*DAA, means direct anterior approach; PLA, posterolateral approach; ROM, Range of Motion*.

### Perioperative Outcomes and Complications

Perioperative blood loss of the DAA group was less than that of the PLA group. In addition, DAA-THA was associated with a shorter hospital stay than PLA-THA (Table 5).

**Table 5:** Surgical characteristics and perioperative results.

	**DAA**	**PLA**	***P*-Value**
	**(*n* = 65)**	**(*n* = 62)**	
Operation time(min)	82.2 ± 34.1	95.0 ± 43.1	0.063
Blood Loss			
Intraoperation	185.8 ± 164.8	251.6 ± 167.7	0.028
Postop day 1	588.3 ± 233.3	768.6 ± 304.0	0.000
Postop day 3	831.1 ± 307.0	1046.2 ± 349.1	0.000
Length of stay	6.4 ± 3.4	7.9 ± 3.4	0.014
Postop LLD	0.4 ± 0.5	0.4 ± 0.5	0.486
Blood transfusion	4	7	0.476

*DAA, direct anterior approach; THA, total hip arthroplasty; ROM means Range of Motion; LLD, Leg Length Discrepancy*.

Fourteen complications (21.5%) occurred in the DAA group vs. 8 (12.9%) in the PLA group. Intraoperative proximal femoral fractures occurred in eight hips (three and five in DAA and PLA groups, respectively). Fractures were directly reduced and fixed using steel wire. Superficial wound complications occurred in 6 hips (three in each group). Lateral femoral cutaneous nerve (LFCN) injuries were observed in 8 hips (12.3%; all the DAA group): 4 hips (50%) of the surgeon's first 33 cases and 4 hips (50%) throughout the surgeon's latter 32 cases. Symptoms in 5 hips (83.3%) were alleviated at the last follow-up (Supplementary Table 5).

## Discussion

Fused or ankylosed hips are highly inconvenient, and studies have shown that THA surgery is an effective and economically efficient therapy for affected patients (1, 3, 4). Converting hip fusion to THA is technically challenging. Although there have been many reported successes, surgical difficulties and trauma are concerning (2, 4). DAA-THA is minimally invasive. It has been assumed that hip deformity and fusion are contraindications for DAA surgery. However, recent knowledge indicates the technique can be performed smoothly in many situations and has potential advantages. To date, few studies have compared outcomes of lateral decubitus DAA and PLA-THA in patients with hip fusion. This study revealed that patients receiving DAA-THA recovered faster than those who underwent PLA-THA, indicating that DAA-THA may be an effective solution for converting fused or ankylosed hips to THAs.

The DAA facilitates rapid rehabilitation because it causes less muscle damage and less bleeding. Postoperatively, patients are quickly able to bear weight and perform functional exercises, and risk of posterior dislocation is low. Rapid recovery after DAA-THA is well known (13–15). Bremer et al. validated this by showing that gluteal muscles in patients who underwent DAA were well preserved via MRI (16). Wu et al. reported that DAA may be successfully used to treat hip fusion (17). Patients who underwent DAA surgery had improved pain, joint motion, and functional evaluation scores. In addition, DAA surgery also includes advantages of preservation of external rotation and abductor muscle strength (18, 19), which is of great significance in fusion of hip joints. On the other hand, a large number of patients undergoing conventional initial total hip replacement have benefited from rapid recovery using the DAA approach. Undoubtedly, this is of huge attraction for doctors and patients in complex cases such as hip fusion. Herein, functional scores of patients undergoing DAA surgery were significantly higher than those of patients undergoing PLA. Interestingly, an analysis of HHS during the same postoperative period revealed that the difference between the two groups was mainly due to functional rather than pain scores, possibly due to the improved functional recovery and the ability to lose crutches or walkers earlier in the recovery period. Therefore, patients who underwent DAA tended to be satisfied with their hips. The largest between-group difference observed was between early OHS scores of DAA and PLA groups, which was likely due to the fact that the scoring system is based on patient self-evaluation. In contrast, OHS is based on more detailed evaluation items, which less significant reflect a ceiling effect than other patient-reported outcome evaluation systems (20).

Previous studies have shown DAA can achieve the same excellent acetabular cup positioning as can be achieved with PLA (21–24). Zhao et al. compared the acetabular position of patients who underwent THA using the DAA or PLA. The DAA was associated with lessened acetabular cup abduction angle fluctuation (25). Herein, prosthesis orientation and alignment differences between groups were not significant. Therefore, we used the modified Lewinneck safety zone standard to evaluate acetabular prosthesis installation, which revealed that more acetabular cups in the DAA group were ideally positioned with respect to anteversion and abduction. This may be related to the fact that the acetabulum is exposed more easily in DAA-THA in the lateral recumbent position. Although deviation was large in the early stages, early clinical outcomes were not affected. This imperfection was quickly eliminated as the surgeon gained experience. Parameters of anterior approach surgery can be quickly stabilized throughout the surgeon's learning curve. In this presented study, the DAA group quickly obtained satisfying radiological outcomes in both extension and flexion fused cases (Figure 5). This statement should be interpreted with caution since whether the “safe zone” proposed by Lewinneck et al. reduces risk of hip dislocation after THA is controversial (10, 26, 27). Compared with other THA patients, those with ankylosing spondylitis had a higher rate of postoperative dislocation due to spinopelvic stiffness (28). In the early cases of ankylotic hip replacement surgery, even in patients undergoing posterior approach surgery, a considerable proportion experienced anterior dislocation (29). For such patients, the “safe zone” was narrow and required more accurate prosthesis positioning.

It was once believed that placing the femoral component in the proper supine position in DAA-THA would be difficult due to problems with elevating the proximal femur (7, 30). It is sometimes necessary to fold the operating table so that the femur is extended to complete this operation. Even so, the extent of hip extension is still limited. However, in the lateral position, the hip joint can be extended without restriction, making it easier than ever to release the posterior joint capsule and expose the proximal femur (8). With DAA in the supine position, the ipsilateral knee is in flexion in order to expose the proximal femur. In the lateral position, the knee joint is straight. This will help relax the tensor fasciae latae and reduce incisional tension. Herein, femoral prosthesis placement of the two groups assessed did not significantly differ.

The following risk factors of hip joint surgery affecting early function have been previously identified: surgical approach, preoperative HHS score, comorbidities (1, 31, 32). Herein, the preoperative fusion type, surgical approach, and early postoperative joint ROM were independent risk factors that affected early postoperative HHS. In a fused hip in flexion, adequate release of the anteriorly contracted capsule via PLA was not an easy job. This stage requires extra care to avoid damage to the main blood vessels so that it is sometimes omitted intentionally. Long-term contracture of soft tissues and inadequate release will cause joint extension limitation in the early postoperative period in some patients, directly resulting in inferior HHS. Early postoperative ROM is likely to mediate the effect of hip fusion type on early postoperative HHS. Limited joint mobility may adversely affect the patient's gait and daily life, further affecting HHS. In contrast, it's easy and safe to release capsular ligament from the front view. This facilitates ROM improvement, even in flexural-fused hips (Supplementary Files Case 2–7, Supplementary Videos 1–3). Joint extension limitation in these patients can be gradually improved with continuous rehabilitation. At the 1-year postoperative follow-up, no significant between-group differences in ROM nor HHS were observed.

Compared with PLA, DAA-THA has obvious advantages in terms of blood loss and length of stay. Better perioperative outcomes verified the superiority of DAA as a minimally invasive surgery. Regarding postoperative complications, no significant between-group difference regarding in the incidence of complications was observed. LFCN injury was a unique complication of DAA. In our study, LFCN occurred 4 of the first 33 and the last 32 hips, indicating that the learning curve of the surgeon did not significantly contribute to postoperative LFCN injury. The position of the incision (33), small femoral offset (34), and sartorius and tensor fasciae latae muscle separation may contribute to LFCN injury (8). At the six-month postsurgical follow-up, LFCN symptoms were relieved in five patients (83.3%). Despite the occurrence of this complication, clinical outcomes of the DAA group were not worse than those of the PLA group. Hence, fear of postoperative LFCN injury should not cause novice surgeons to avoid selecting the DAA approach.

This study has several limitations. First, it was a retrospective study, patients were not randomly assigned to two groups. The early cases in this cohort are mainly using PLA, while the later cases are DAA since we became familiar with this approach (Supplementary Figure 2). However, the rarity of fused hip cases makes conducting a large-scale, randomized controlled trial impractical. When analyzing the characteristics of cases retrospectively, we set up strict inclusion and exclusion criteria to minimize selection bias. There is no difference between the baseline data of the two groups of patients. Second, since the surgical technique is relatively new, long-term clinical results remain unavailable. Given our limited knowledge of hip fusion treatment with DAA-THA, results of this study are meaningful. Mid-and long-term follow-up will be performed to further assess the effect of this technique in the future. Especially for patients with extended hip fusion (because the proximal femur leans forward), DAA-THA facilitates the release of external rotators and the posterior capsule to expose the proximal femur.

## Conclusions

For patients with complex hip fusion, lateral decubitus DAA-THA may be an ideal option if supine DAA is difficult due to a lack of dedicated operating equipment. Compared with posterolateral THA, this technique seems to yield satisfactory clinical and radiological results.

## Data Availability Statement

All relevant data are included in the report and its associated files.

## Ethics Statement

The studies involving human participants were reviewed and approved by Ethics Committee of the First Affiliated Hospital of USTC. The patients/participants provided their written informed consent to participate in this study. Written informed consent was obtained from the individual(s) for the publication of any potentially identifiable images or data included in this article.

## Author Contributions

XZ: conceptualization, and methodology. JD and LK: formal analysis, and investigation. JD: writing—original draft preparation. SZ: writing—review and editing. CZ: resources. JW, CZ, and XS: supervision. All authors contributed to the article and approved the submitted version.

## Funding

This work was supported by the National Natural Science Foundation of China (Grant No. 81871788), the Key Research and Development Program of Anhui Province (No. 202004j07020013), the project for Science and Technology leader of Anhui Province (Grant No. 2018H177), the Anhui Provincial Postdoctoral Science Foundation (Grant No. 2019B302), the Scientific Research Fund of Anhui Education Office (Grant No.2020jyxm2316), the National Natural Science Foundation of Anhui Province(2108085QH319), and the Fundamental Research Funds for the Central Universities (WK9110000173).

## Conflict of Interest

The authors declare that the research was conducted in the absence of any commercial or financial relationships that could be construed as a potential conflict of interest.

## Publisher's Note

All claims expressed in this article are solely those of the authors and do not necessarily represent those of their affiliated organizations, or those of the publisher, the editors and the reviewers. Any product that may be evaluated in this article, or claim that may be made by its manufacturer, is not guaranteed or endorsed by the publisher.

## Abbreviations

CI: Confidence interval
DAA: Direct anterior approach
HHS: Harris Hip Score
LFCN: Lateral femoral cutaneous nerve
OHS: Oxford Hip Score
PLA: Posterolateral approach
ROM: Range of motion
THA: Total hip arthroplasty.

## References

[1] Abe H Sakai T Takao M Nishii T Nakamura N Sugano N. Difference in stem alignment between the direct anterior approach and the posterolateral approach in total hip arthroplasty. J Arthroplasty. (2015) 30:1761–6. 10.1016/j.arth.2015.04.026 25956522

[2] Awad ME Farley BJ Mostafa G Saleh KJ. Direct anterior approach has short-term functional benefit and higher resource requirements compared with the posterior approach in primary total hip arthroplasty : a meta-analysis of functional outcomes and cost. Bone Joint J. (2021) 103–B:1078–87. 10.1302/0301-620X.103B6.BJJ-2020-1271.R1 34058867

[3] Ayekoloye CI Abu Qa'oud M Radi M Leon SA Kuzyk P Safir O. Review of complications, functional outcome, and long-term survival following conversion of hip fusion to total hip arthroplasty. Bone Joint J. (2021) 103–B(7 Supple B):129–34. 10.1302/0301-620X.103B7.BJJ-2020-2382.R1 34192904

[4] Bremer AK Kalberer F Pfirrmann CW Dora C. Soft-tissue changes in hip abductor muscles and tendons after total hip replacement: comparison between the direct anterior and the transgluteal approaches. J Bone Joint Surg Br. (2011) 93:886–9. 10.1302/0301-620X.93B7.25058 21705558

[5] Chen M Luo Z Ji X Cheng P Tang G Shang X. Direct anterior approach for total hip arthroplasty in the lateral decubitus position: our experiences and early results. J Arthroplasty. (2017) 32:131–8. 10.1016/j.arth.2016.05.066 27369300

[6] Cheng TE Wallis JA Taylor NF Holden CT Marks P Smith CL. A prospective randomized clinical trial in total hip arthroplasty-comparing early results between the direct anterior approach and the posterior approach. J Arthroplasty. (2017) 32:883–90. 10.1016/j.arth.2016.08.027 27687805

[7] Christensen CP Jacobs CA. Comparison of patient function during the first six weeks after direct anterior or posterior total hip arthroplasty (THA): a randomized study. J Arthroplasty. (2015) 30(9 Suppl):94–7. 10.1016/j.arth.2014.12.038 26096071

[8] Danoff JR Bobman JT Cunn G Murtaugh T Gorroochurn P Geller JA. Redefining the acetabular component safe zone for posterior approach total hip arthroplasty. J Arthroplasty. (2016) 31:506–11. 10.1016/j.arth.2015.09.010 26461487

[9] Dorr LD Callaghan JJ. Death of the Lewinnek “Safe Zone”. J Arthroplasty. (2019) 34:1–2. 10.1016/j.arth.2018.10.035 30527340

[10] Fernandez-Fairen M Murcia-Mazón A Torres A Querales V Murcia A Jr. Is total hip arthroplasty after hip arthrodesis as good as primary arthroplasty? Clin Orthop Relat Res. (2011) 469:1971–83. 10.1007/s11999-010-1704-y 21116751 PMC3111784

[11] Flecher X Ollivier M Maman P Pesenti S Parratte S Argenson JN. Long-term results of custom cementless-stem total hip arthroplasty performed in hip fusion. Int Orthop. (2018) 42:1259–64. 10.1007/s00264-018-3762-9 29352333

[12] Flevas DA Tsantes AG Mavrogenis AF. Direct anterior approach total hip arthroplasty revisited. JBJS Rev. (2020) 8:e0144. 10.2106/JBJS.RVW.19.00144 32304500

[13] Gautam D Malhotra R. Total hip arthroplasty in ankylosing spondylitis with extension contracture of hips. J Arthroplasty. (2019) 34:71–6. 10.1016/j.arth.2018.08.025 30342954

[14] Grappiolo G Bruno CF Loppini M Mercurio M Castioni D Gasparini G. Conversion of fused hip to total hip arthroplasty: long-term clinical and radiological outcomes. J Arthroplasty. (2021) 36:1060–6. 10.1016/j.arth.2020.09.030 33082070

[15] Jacobs CA Kusema ET Keeney BJ Moschetti WE. Does the thigh circumference affect the positioning of the acetabular component when using the direct anterior approach in total hip arthroplasty? Bone Joint J. (2019) 101–B:529–35. 10.1302/0301-620X.101B5.BJJ-2018-0847.R2 31038997

[16] Jia F Guo B Xu F Hou Y Tang X Huang L. comparison of clinical radiographic and surgical outcomes of total hip arthroplasty between direct anterior and posterior approaches: a systematic review and meta-analysis. Hip Int. (2019) 29:584–96. 10.1177/1120700018820652 30595060

[17] Katakam A Bedair HS Melnic CM. Do all rigid and unbalanced spines present the same risk of dislocation after total hip arthroplasty? A comparison study between patients with ankylosing spondylitis and history of spinal fusion. J Arthroplasty. (2020) 35:3594–600. 10.1016/j.arth.2020.06.048 32660797

[18] Lewinnek GE Lewis JL Tarr R Compere CL Zimmerman JR. Dislocations after total hip-replacement arthroplasties. J Bone Joint Surg Am. (1978) 60:217–20. 10.2106/00004623-197860020-00014 641088

[19] Lin TJ Bendich I Ha AS Keeney BJ Moschetti WE Tomek IM. Comparison of radiographic outcomes after total hip arthroplasty between the posterior approach and direct anterior approach with intraoperative fluoroscopy. J Arthroplasty. (2017) 32:616–23. 10.1016/j.arth.2016.07.046 27612607 PMC5258737

[20] Malek IA Royce G Bhatti SU Whittaker JP Phillips SP Wilson IR. A comparison between the direct anterior and posterior approaches for total hip arthroplasty: the role of an 'Enhanced Recovery' pathway. Bone Joint J. (2016) 98–B:754–60. 10.1302/0301-620X.98B6.36608 27235516

[21] Mannion AF Nauer S Arsoy D Impellizzeri FM Leunig M. The association between comorbidity and the risks and early benefits of total hip arthroplasty for hip osteoarthritis. J Arthroplasty. (2020) 35:2480–7. 10.1016/j.arth.2020.04.090 32466998

[22] Meermans G Konan S Das R Volpin A Haddad FS. The direct anterior approach in total hip arthroplasty: a systematic review of the literature. Bone Joint J. (2017) 99–b:732–40. 10.1302/0301-620X.99B6.38053 28566391

[23] Melman WP Mollen BP Kollen BJ Verheyen CC. First experiences with the direct anterior approach in lateral decubitus position: learning curve and 1 year complication rate. Hip Int. (2015) 25:251–7. 10.5301/hipint.5000221 25684251

[24] Nadler SB Hidalgo JH Bloch T. Prediction of blood volume in normal human adults. Surgery. (1962) 51:224–32. 21936146

[25] Ostendorf M van Stel HF Buskens E Schrijvers AJ Marting LN Verbout AJ. Patient-reported outcome in total hip replacement. A comparison of five instruments of health status. J Bone Joint Surg Br. (2004) 86:801–8. 10.1302/0301-620X.86B6.14950 15330018

[26] Ozaki Y Homma Y Sano K Baba T Ochi H Desroches A. Small femoral offset is a risk factor for lateral femoral cutaneous nerve injury during total hip arthroplasty using a direct anterior approach. Orthop Traumatol Surg Res. (2016) 102:1043–7. 10.1016/j.otsr.2016.08.019 27777087

[27] Reininga IH Stevens M Wagenmakers R Boerboom AL Groothoff JW Bulstra SK. Comparison of gait in patients following a computer-navigated minimally invasive anterior approach and a conventional posterolateral approach for total hip arthroplasty: a randomized controlled trial. J Orthop Res. (2013) 31:288–94. 10.1002/jor.22210 22886805

[28] Rudin D Manestar M Ullrich O Erhardt J Grob K. The anatomical course of the lateral femoral cutaneous nerve with special attention to the anterior approach to the hip joint. J Bone Joint Surg Am. (2016) 98:561–7. 10.2106/JBJS.15.01022 27053584

[29] Sponseller PD McBeath AA Perpich M. Hip arthrodesis in young patients. A long-term follow-up study. J Bone Joint Surg Am. (1984) 66:853–9. 10.2106/00004623-198466060-00005 6234319

[30] Stolarczyk A Stolarczyk M Stepinski P Dorocinska MK Swiercz M Szymczak J. The direct anterior approach to primary total hip replacement: radiological analysis in comparison to other approaches. J Clin Med. (2021) 10:2246. 10.3390/jcm10112246 34064295 PMC8196856

[31] Tang WM Chiu KY. Primary total hip arthroplasty in patients with ankylosing spondylitis. J Arthroplasty. (2000) 15:52–8. 10.1016/S0883-5403(00)91155-0 10654462

[32] Tezuka T Heckmann ND Bodner RJ Dorr LD. Functional safe zone is superior to the lewinnek safe zone for total hip arthroplasty: why the lewinnek safe zone is not always predictive of stability. J Arthroplasty. (2019) 34:3–8. 10.1016/j.arth.2018.10.034 30454867

[33] Wu H Cheng WD Jing J. Total hip arthroplasty by direct anterior approach in the lateral position for the treatment of ankylosed hips. Eur J Orthop Surg Traumatol. (2020) 30:993–1001. 10.1007/s00590-020-02655-w 32185574

[34] Zhao HY Kang PD Xia YY Shi XJ Nie Y Pei FX. Comparison of early functional recovery after total hip arthroplasty using a direct anterior or posterolateral approach: a randomized controlled trial. J Arthroplasty. (2017) 32:3421–8. 10.1016/j.arth.2017.05.056 28662957

