# Supplementary material for: Conversion of a Fused or Ankylosed Hip to Total Hip Arthroplasty: Is the Direct Anterior Approach in the Lateral Decubitus Position an Ideal Solution?
Source: Front Surg. 2022 Feb 8;9:819530. doi: 10.3389/fsurg.2022.819530 (PMC8861463; doi:10.3389/fsurg.2022.819530)
Supplement: Supplementary file 9 [file Presentation_1.PDF]

Case 1

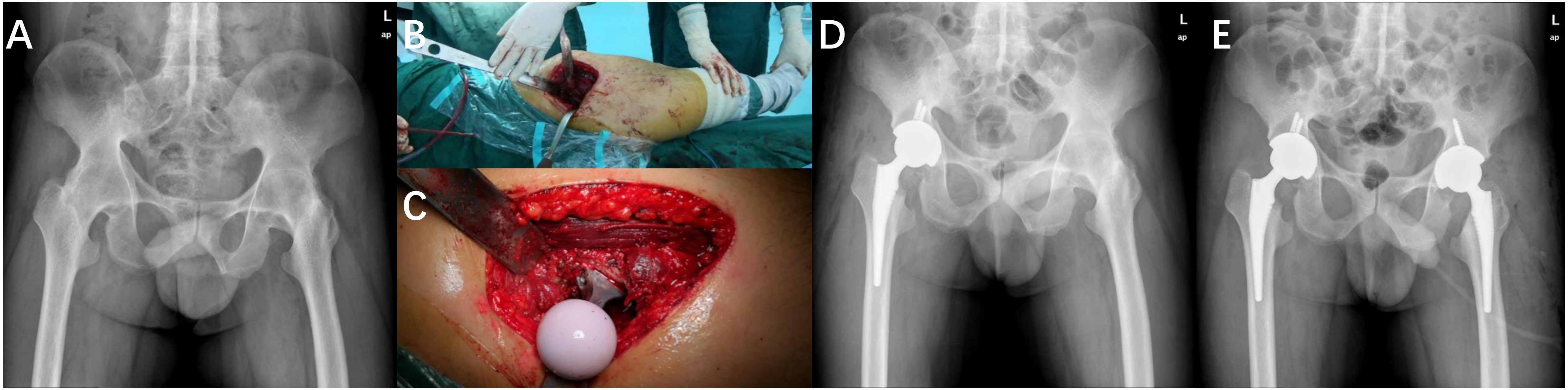

Conversion of extended hip fusion to DAA-THA in a 29-year-old male. Preoperative (A), and postoperative(D,E) radiographs. B,C)Intraoperative photographs. (B) Exposing the proximal femur.(C) Implanting the femoral component.
