# Supplementary material for: Conversion of a Fused or Ankylosed Hip to Total Hip Arthroplasty: Is the Direct Anterior Approach in the Lateral Decubitus Position an Ideal Solution?
Source: Front Surg. 2022 Feb 8;9:819530. doi: 10.3389/fsurg.2022.819530 (PMC8861463; doi:10.3389/fsurg.2022.819530)
Supplement: Supplementary file 10 [file Presentation_2.PDF]

Case 2

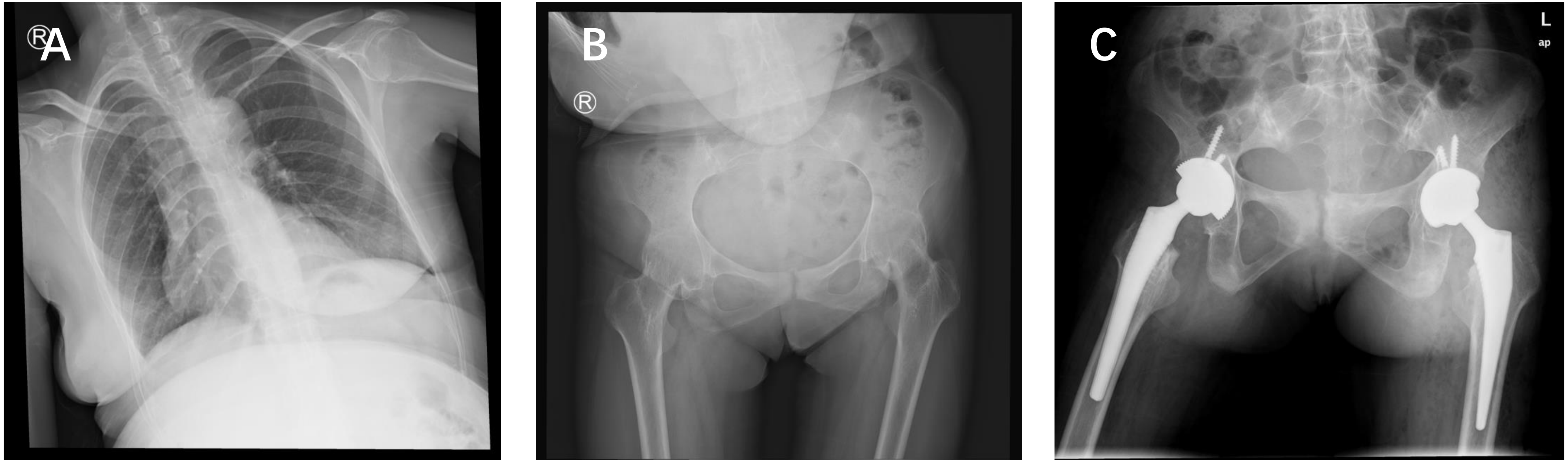

Conversion of flexural hip fusion to DAA-THA in a 39-year-old female. Preoperative (A,B), and 1 month postoperative(C) radiographs.

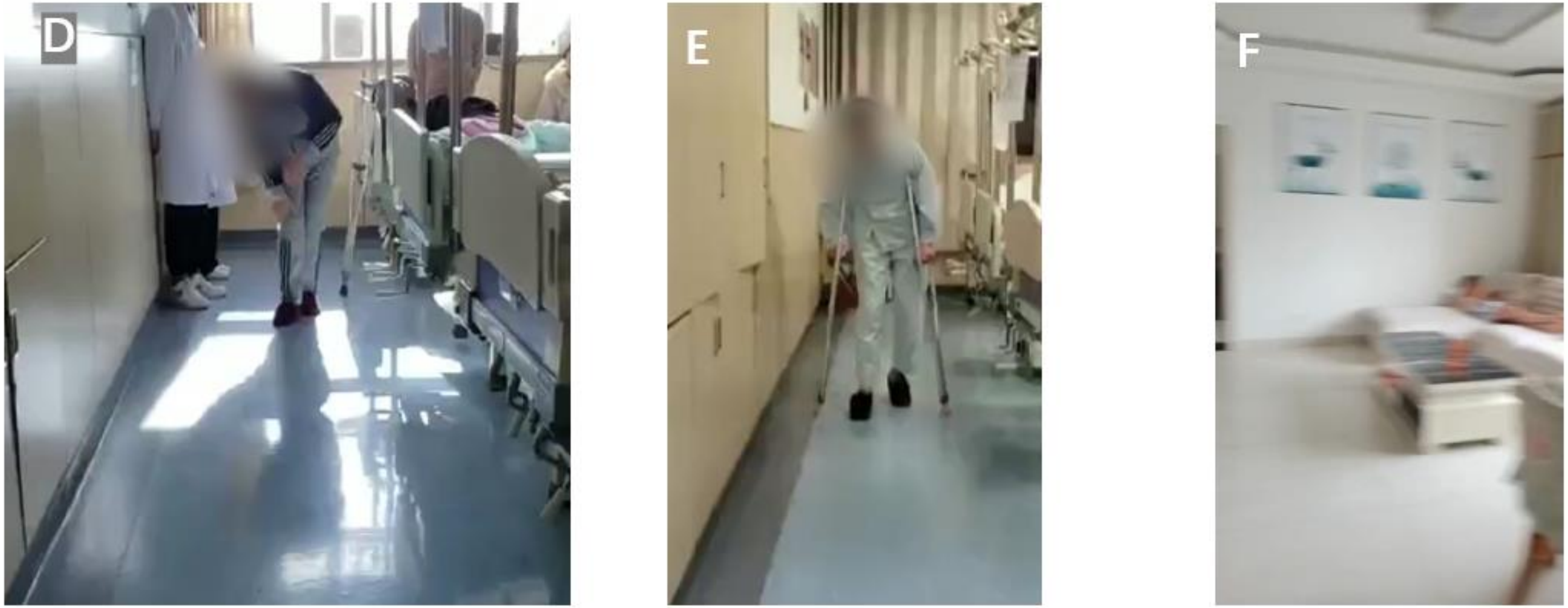

Preoperative(D,video1), postoperative(E,video2), and 1 month(F,video3) postoperative videos.

Case 3

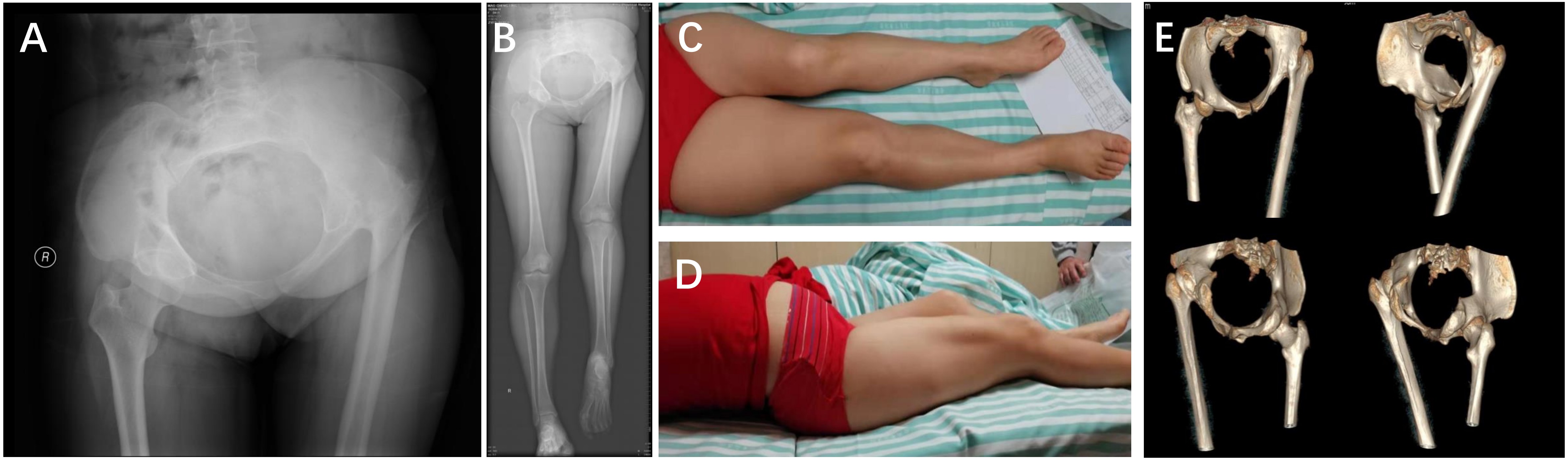

Conversion of flexural hip fusion to DAA-THA in a 41-year-old female. (A,B) Preoperative radiographs.(C,D) Preoperative ankylosis of hip. (E) Preoperative 3d CT reconstructions.

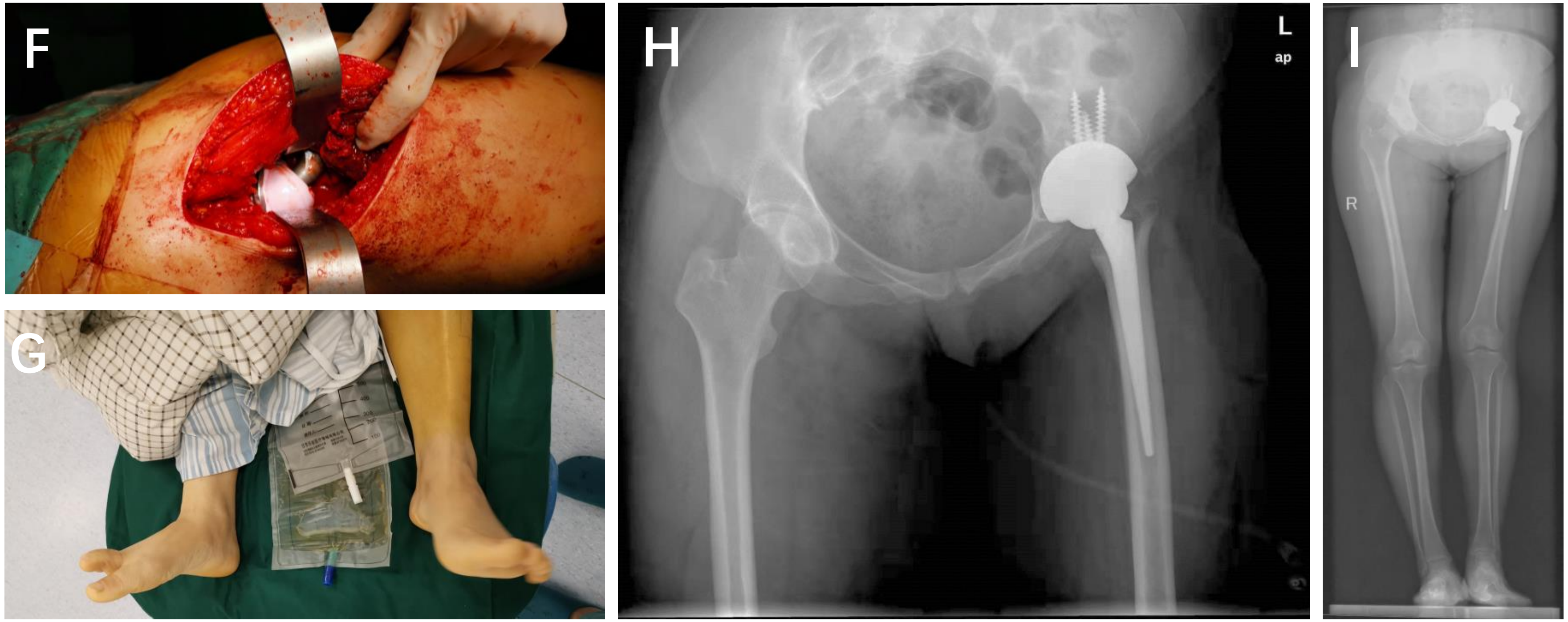

F,G)Intraoperative photographs. (F) Implanting the femoral component.(G)Assessing restoration of limb length. Postoperative(H) and 1 month (I) postoperative radiographs.

Case 4

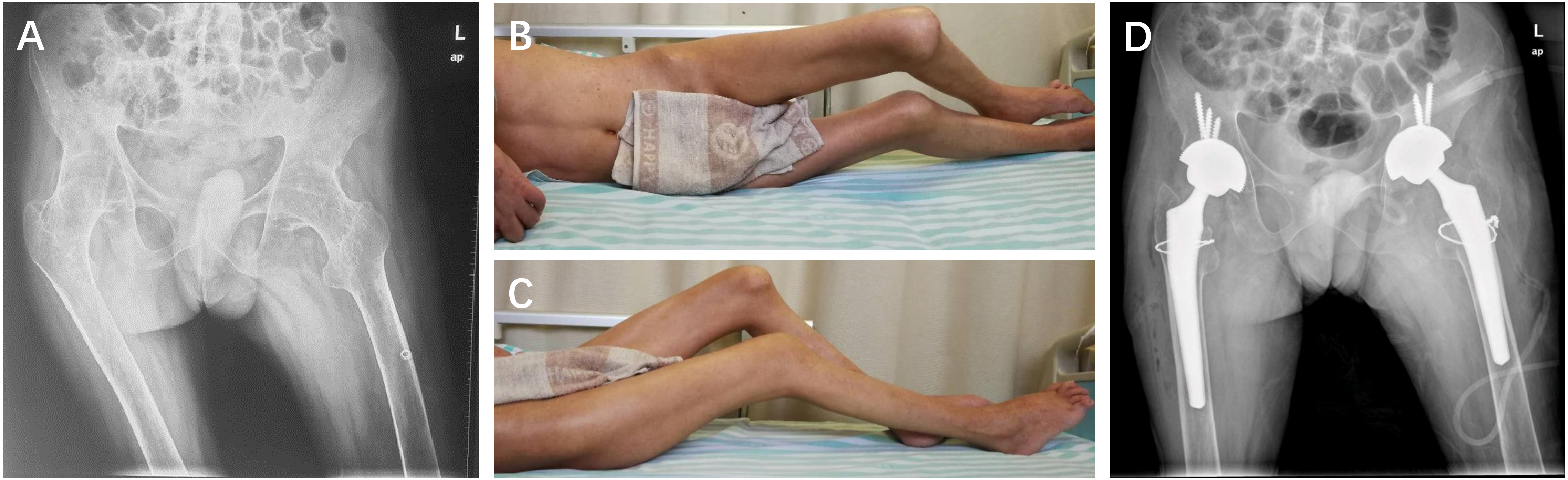

Conversion of flexural hip fusion to DAA-THA in a 37-year-old male. Preoperative (A), and postoperative(D) radiographs. (B)and(C) show the ankylosis of hip preoperatively.

Case 5

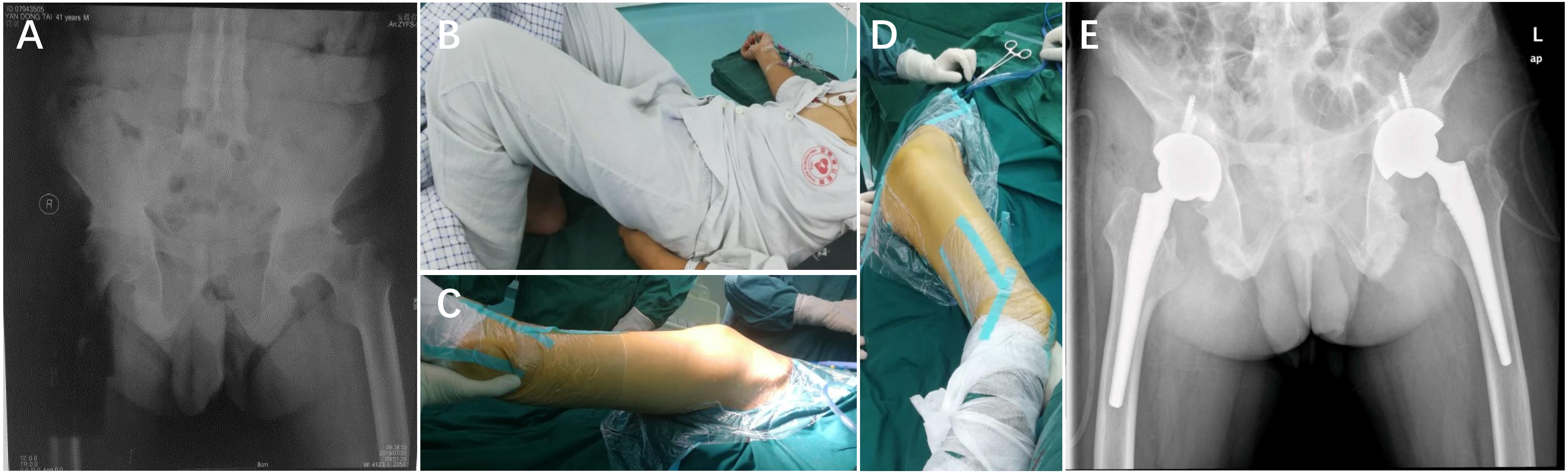

Conversion of flexural hip fusion to DAA-THA in a 41-year-old male. Preoperative (A), and postoperative(E) radiographs. (B), (C), and(D) show the ankylosis of hip preoperatively.

Case 6

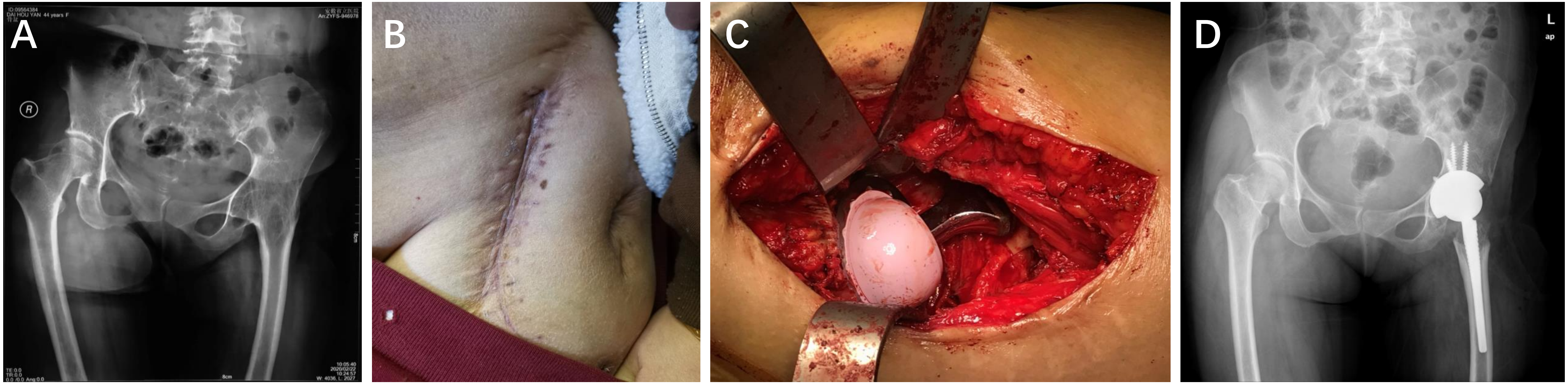

Conversion of flexural hip fusion to DAA-THA in a 44-year-old female. Preoperative (A), and postoperative(D) radiographs. Preoperative(B), and intraoperative(C) photographs of a fused hip.
