# Supplementary material for: Conversion of a Fused or Ankylosed Hip to Total Hip Arthroplasty: Is the Direct Anterior Approach in the Lateral Decubitus Position an Ideal Solution?
Source: Front Surg. 2022 Feb 8;9:819530. doi: 10.3389/fsurg.2022.819530 (PMC8861463; doi:10.3389/fsurg.2022.819530)
Supplement: Supplementary file 11 [file Presentation_3.PDF]

Case 7

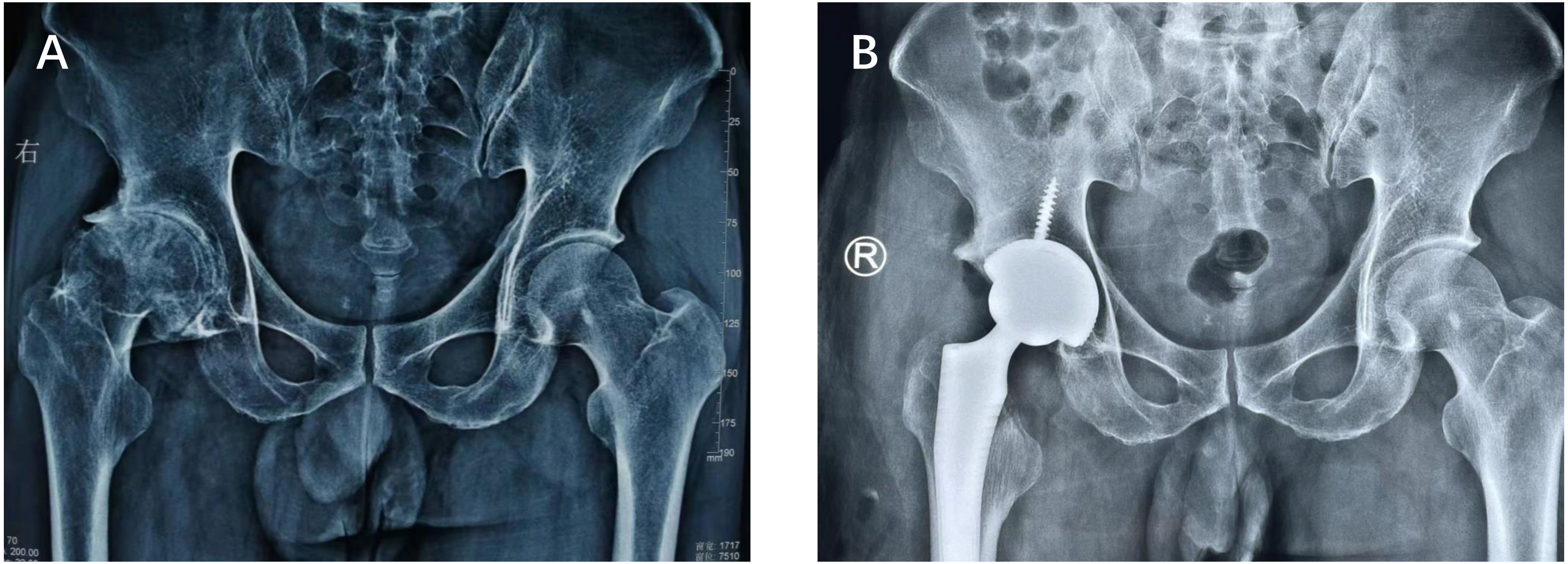

Conversion of stiff hip to DAA-THA in a 46-year-old male performed by a relatively junior doctor (ZXZ, practicing as an arthroplasty surgeon for 5 years). Preoperative (A) and 1 month postoperative(B) radiographs.
