# Supplementary material for: Conversion of a Fused or Ankylosed Hip to Total Hip Arthroplasty: Is the Direct Anterior Approach in the Lateral Decubitus Position an Ideal Solution?
Source: Front Surg. 2022 Feb 8;9:819530. doi: 10.3389/fsurg.2022.819530 (PMC8861463; doi:10.3389/fsurg.2022.819530)
Supplement: Supplementary file 12 [file Data_Sheet_1.DOCX]

**Supplementary files**

**Supplementary figure**





**Supplementary Fig. 1** Harris sccore ranks of two groups for 1-month follow-up

**
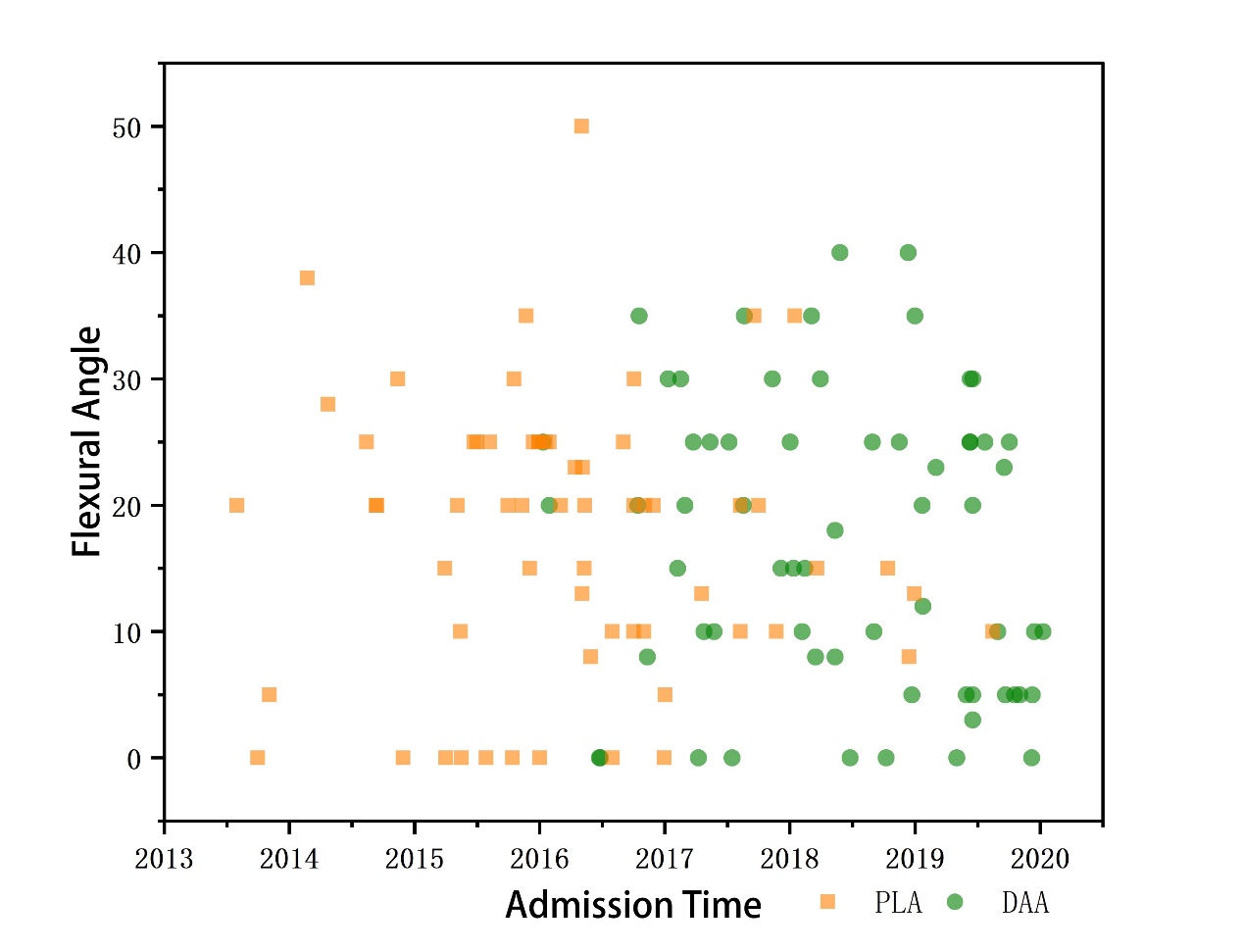
**

**Supplementary Fig. 2** Adimission time and flexural angle of each patient.

**Supplementary tables**

Supplementary Table.1 Harris scores of two groups for 1-month to 1-year follow-up

|  | DAA | PLA | P-Value |
| --- | --- | --- | --- |
|  | （n=65) | （n=62) |  |
| HHS at 1-month | 71.8±8.3 | 64.5±6.7 | 0.000 |
| Pain | 32.0±6.3 | 30.5±6.1 | 0.171 |
| Function | 39.8±4.7 | 34.0±4.7 | 0.000 |
| Gait | 9.6±1.5 | 10.0±1.4 | 0.115 |
| Walking aid | 9.9±1.9 | 7.2±2.2 | 0.000 |
| Distance | 5.8±1.5 | 4.2±1.3 | 0.000 |
| Life | 7.5±1.8 | 5.6±1.8 | 0.000 |
| Malformation | 4 | 4 |  |
| ROM | 3.0±0.2 | 3.0±0.1 | 0.591 |
| Without walking aid | 39(60.0%) | 13(21.0%) | 0.000 |
| HHS at 3-month | 81.6±6.7 | 77.8±4.2 | 0.000 |
| Pain | 37.9±5.3 | 37.4±4.4 | 0.600 |
| Function | 43.7±4.1 | 40.4±4.2 | 0.000 |
| Gait | 9.9±1.5 | 9.9±1.4 | 0.867 |
| Walking aid | 10.6±1.2 | 9.9±1.9 | 0.007 |
| Distance | 6.3±1.7 | 5.2±1.9 | 0.001 |
| Life | 8.4±2.1 | 7.0±2.3 | 0.000 |
| Malformation | 4 | 4 |  |
| ROM | 4.4±0.5 | 4.4±0.5 | 0.965 |
| Without walking aid | 59(90.8%) | 45（72.6%） | 0.008 |
| HHS at 1-year | 85.4±5.5 | 85.1±4.3 | 0.731 |
| Pain | 39.6±4.7 | 40.3±4.4 | 0.376 |
| Function | 45.8±4.0 | 44.8±3.3 | 0.121 |
| Gait | 10.2±1.3 | 10.2±1.3 | 0.873 |
| Walking aid | 10.8±0.8 | 10.7±1.0 | 0.653 |
| Distance | 6.3±1.6 | 6.0±1.4 | 0.305 |
| Life | 9.6±2.3 | 9.0±2.3 | 0.149 |
| Malformation | 4 | 4 |  |
| ROM | 4.9±0.3 | 4.9±0.3 | 0.492 |
| Without walking aid | 62(95.4%) | 58(93.5%) | 0.949 |

DAA, means direct anterior approach; PLA, posterolateral approach; HHS, Harris Hip Score.

Supplementary Table.2 Hip Range of Motion at the 1-year follow-up

| ROM | DAA | PLA | P-Valuea |
| --- | --- | --- | --- |
|  | （n=65) | （n=62) |  |
| Flexion | 98.7±9.2 | 100.1±7.8 | 0.357 |
| Extension | 5.4±3.4 | 4.6±3.9 | 0.197 |
| Adduction | 26.9±4.5 | 26.2±4.4 | 0.348 |
| Abduction | 34.1±6.5 | 33.0±6.1 | 0.354 |
| Internal Rotation | 30.9±5.2 | 31.2±4.8 | 0.748 |
| External Rotation | 29.1±5.2 | 28.6±5.1 | 0.625 |

DAA, direct anterior approach; THA, total hip arthroplasty; ROM, Range of Motion.

Supplementary Table.3 The mediating effect analysis of ROM

| Outcome variable | Predictive variable | B | t | P-Value | 95%CI | | R^2^ | F |
| --- | --- | --- | --- | --- | --- | --- | --- | --- |
|  |  |  |  |  | LLCI | ULCI |  |  |
| HHS | Fuse position | -0.533 | -3.290 | 0.001 | -0.853 | -0.212 | 0.263 | 8.617 |
|  | Surgical approach | -0.412 | -5.246 | 0.000 | -0.567 | -0.257 |  |  |
|  | Gender | 0.044 | 0.524 | 0.601 | -0.121 | 0.208 |  |  |
|  | Age | 0.170 | 1.631 | 0.106 | -0.036 | 0.375 |  |  |
|  | course | -0.132 | -1.250 | 0.214 | -0.342 | 0.077 |  |  |
| ROM | Fuse position | -0.461 | -2.791 | 0.006 | -0.788 | -0.134 | 0.233 | 7.356 |
|  | Surgical approach | -0.335 | -4.181 | 0.000 | -0.493 | -0.176 |  |  |
|  | Gender | -0.170 | -2.008 | 0.047 | -0.337 | -0.002 |  |  |
|  | Age | 0.100 | 0.941 | 0.348 | -0.110 | 0.310 |  |  |
|  | course | 0.135 | 1.247 | 0.215 | -0.079 | 0.348 |  |  |
| HHS | Fuse position | -0.427 | -2.618 | 0.010 | -0.750 | -0.104 | 0.303 | 8.691 |
|  | ROM | 0.229 | 2.635 | 0.010 | 0.057 | 0.402 |  |  |
|  | Surgical approach | -0.335 | -4.087 | 0.000 | -0.498 | -0.173 |  |  |
|  | Gender | 0.082 | 1.001 | 0.319 | -0.081 | 0.246 |  |  |
|  | Age | 0.147 | 1.440 | 0.153 | -0.055 | 0.348 |  |  |
|  | course | -0.163 | -1.569 | 0.119 | -0.369 | 0.043 |  |  |

DAA, means direct anterior approach; PLA, posterolateral approach; HHS, Harris Hip Score; ROM, Range of Motion; LLCI, Lower Level of Confidence Interval; ULCI, Upper Level of Confidence Interval.

Supplementary Table.4 The mediating effect of ROM in the relationship between fused position and Harris scores for 1 month follow-up

|  | Effect | BootSE | BootLLCI | BootULCI | Relative effect |
| --- | --- | --- | --- | --- | --- |
| Total | -0.533 | 0.156 | -0.828 | -0.220 |  |
| Direct | -0.427 | 0.157 | -0.719 | -0.104 | 0.802 |
| Indirect | -0.106 | 0.057 | -0.234 | -0.016 | 0.198 |

BootSE, Bootstrap Standard Error; BootLLCI, Bootstrap Lower Level of Confidence Interval; BootULCI, Bootstrap Upper Level of Confidence Interval.

Supplementary Table.5 Complications

|  | DAA | PLA | P-Valueb |
| --- | --- | --- | --- |
|  | （n=65) | （n=62) |  |
| Total | 14 | 8 | 0.199 |
| Intraoperative proximal femoral fracture | 3（4.6%） | 5（8.1%） | 0.664 |
| Superficial wound complications | 3（4.6%） | 3（4.8%） | 1.000 |
| Deep infection | 0（0%） | 0（0%） | 1.000 |
| Deep vein thrombosis | 0（0%） | 0（0%） | 1.000 |
| Dislocation | 0（0%） | 0（0%） | 1.000 |
| LFCN palsy | 8（12.3%） | 0（0%） | 0.000 |

DAA, direct anterior approach; THA, total hip arthroplasty; LFCN, lateral femoral cutaneous nerve.
